# Dissolution Advantage of Nitazoxanide Cocrystals in the Presence of Cellulosic Polymers

**DOI:** 10.3390/pharmaceutics12010023

**Published:** 2019-12-25

**Authors:** Reynaldo Salas-Zúñiga, Christian Rodríguez-Ruiz, Herbert Höpfl, Hugo Morales-Rojas, Obdulia Sánchez-Guadarrama, Patricia Rodríguez-Cuamatzi, Dea Herrera-Ruiz

**Affiliations:** 1 Facultad de Farmacia, Universidad Autónoma del Estado de Morelos, Av. Universidad 1001, Cuernavaca 62209, Mexico; 2 Centro de Investigaciones Químicas, Instituto de Investigación en Ciencias Básicas y Aplicadas, Universidad Autónoma del Estado de Morelos, Av. Universidad 1001, Cuernavaca 62209, Mexico; 3 Departamento de Ingeniería Química, Universidad Politécnica de Tlaxcala, Carretera Federal Tlaxcala-Puebla Km 9.5, Tepeyanco, Tlaxcala 90180, Mexico

**Keywords:** nitazoxanide, cocrystals, multicomponent crystals, dissolution behavior, supersaturated formulations, crystallization inhibitors, drug-polymer interactions

## Abstract

The effect of hydroxypropyl methylcellulose (HPMC) and methylcellulose (Methocel^®^ 60 HG) on the dissolution behavior of two cocrystals derived from nitazoxanide (NTZ), viz., nitazoxanide-glutaric acid (NTZ-GLU, 1:1) and nitazoxanide-succinic acid (NTZ-SUC, 2:1), was explored. Powder dissolution experiments under non-sink conditions showed similar dissolution profiles for the cocrystals and pure NTZ. However, pre-dissolved cellulosic polymer in the phosphate dissolution medium (pH 7.5) modified the dissolution profile of NTZ when starting from the cocrystals, achieving transient drug supersaturation. Subsequent dissolution studies under sink conditions of polymer-based pharmaceutical powder formulations with NTZ-SUC cocrystals gave a significant improvement of the apparent solubility of NTZ when compared with analogous formulations of pure NTZ and the physical mixture of NTZ and SUC. Scanning electron microscopy and powder X-ray diffraction analysis of samples recovered after the powder dissolution studies showed that the cocrystals undergo fast dissolution, drug supersaturation and precipitation both in the absence and presence of polymer, suggesting that the solubilization enhancement is due to polymer-induced delay of nucleation and crystal growth of the less soluble NTZ form. The study demonstrates that the incorporation of an appropriate excipient in adequate concentration can be a key factor for inducing and maintaining the solubilization of poorly soluble drugs starting from co-crystallized solid forms. In such a way, cocrystals can be suitable for the development of solid dosage forms with improved bioavailability and efficacy in the treatment of important parasitic and viral diseases, among others.

## 1. Introduction

**Scheme 1:**
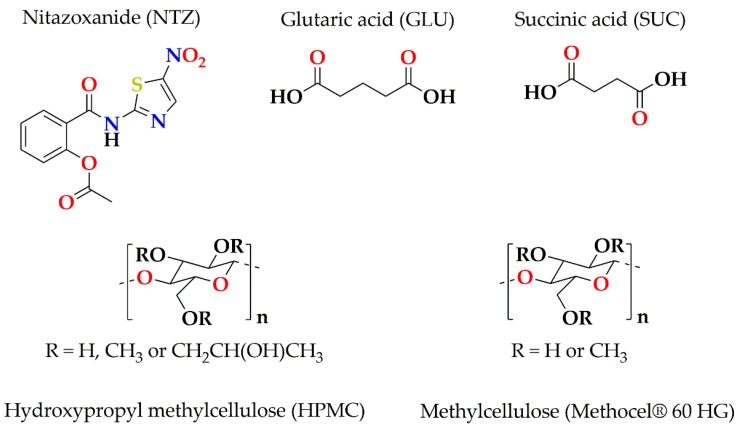
Molecular structures of nitazoxanide (NTZ), coformers and cellulosic polymers used herein to achieve a polymer-based pharmaceutical powder formulation.

Nitazoxanide (NTZ), 2-acetyloxy-*N*-(5-nitro-2-thiazolyl)benzamide, is a synthetic nitrothiazole derivative (Scheme 1) with a broad spectrum of applications as an antiparasitic, antibacterial and antiviral agent, being effective against protozoal infections, helminths, gram negative and gram positive bacteria, and diverse viruses (respiratory viruses, rotavirus, norovirus, coronavirus, hepatitis B and C, dengue-2, yellow fever, Japanese encephalitis, and human immunodeficiency viruses) [1,2,3]. Moreover, NTZ has shown anticancer activity [4], suppresses the production of interleukins (IL) such as IL-6 [5], and is a promising compound for the treatment of neuropathic pain and the Ebola virus disease [6,7]. However, due to poor aqueous solubility (0.0075 mg/mL), NTZ has low bioavailability and requires high doses for treatment [8]. Considering its high permeability across intestinal epithelium [9], NTZ is a class II drug according to the criteria established by the Biopharmaceutical Classification System (BCS). To take advantage of the broad therapeutic spectrum of NTZ, the implementation of strategies to modify its solubility are necessary. A general strategy to overcome the limited solubility of drugs is the generation of novel solid phases, such as metastable amorphous, polymorphs, salts and, more recently, cocrystals [10,11,12].

A cocrystal constitutes a solid single-phase crystalline material composed of two or more molecular and/or ionic compounds in a stoichiometric ratio, which is neither a solvate nor a simple salt. If at least one of the components is an active pharmaceutical ingredient (API) and if the coformer is pharmaceutically acceptable, then the substance is recognized as pharmaceutical cocrystal. A pharmaceutical cocrystal can offer multiple benefits for physicochemical and biopharmaceutical properties of APIs, such as increased solubility and dissolution rates, improvement of mechanical properties, and stability [13,14,15]. Particularly, cocrystals have exhibited higher, similar or lower solubility compared to the parent API, depending on the nature of the coformer and the solution conditions [16,17]. Solubility advantages of cocrystals over parent drugs can be higher by orders of magnitude, however, this also represents a risk due to precipitation to the less soluble form of the drug at supersaturated concentrations reached during the dissolution process [18,19].

Due to this former behavior, cocrystals are potential candidates for the development of supersaturating drug delivery systems [20,21]. A strategy to control cocrystal dissolution, drug supersaturation and precipitation is the use of supersaturated formulations, which incorporates additives and excipients such as cyclodextrins, surfactants and polymers [22]. In such systems, the supersaturation state must be maintained over a reasonable time period in order to promote adequate absorption for increased bioavailability. In this line, there are several recent reports on the effect of polymers and surfactants in increasing solubility and dissolution rates of poorly soluble drugs, starting from a cocrystalline solid phase. Some representative APIs and nutraceutical compounds studied under this approach are celecoxib [23], danazol [24], indomethacin [25], carbamazepine [26,27,28,29,30,31], flufenamic acid [32], cilostazol [33], resveratrol [34], dihydromyricetin [35], tadalafil [36], exemestane [37] and posaconazole [38].

Dissolution methods under non-sink conditions are commonly used for evaluating the ability of poorly soluble APIs to generate and to maintain a supersaturation state in solution [24,39]. Additionally, powder dissolution experiments are a good alternative to intrinsic dissolution tests for cocrystals undergoing rapid transformation to a less soluble phase of the drug [24], as recently found in the biopharmaceutical characterization of cocrystals with meloxicam, indomethacin, apixaban and myricetin [40,41,42,43].

Recently, the synthesis and characterization of NTZ cocrystals using succinic, glutaric, 2,5-dihydroxybenzoic, *p*-aminobenzoic and *p*-aminosalicylic acid as coformers were reported [8,44]. In both publications, improved intrinsic dissolution rates were documented, showing that NTZ cocrystals can improve the solubilization of NTZ in physiologically relevant media [8], thus being good candidates for the future development of novel dosage forms. Herein, two cocrystals, viz., NTZ-GLU and NTZ-SUC (GLU = glutaric acid; SUC = succinic acid), were chosen to evaluate the effect of two cellulosic polymers, namely hydroxypropyl methylcellulose (HPMC) and methylcellulose (Methocel^®^ 60 HG) (Scheme 1), on the dissolution performance of the API, in comparison with pure NTZ, a commercially available NTZ medicine and the respective physical mixtures of NTZ and the coformer. Our study includes an API widely used in treatments concerning tropical neglected diseases, even though scarcely studied [45]. The purpose of this work was to study the behavior of NTZ cocrystals in solution and the conditions to sustain supersaturation levels since these conditions cannot be apparent and must be studied case-by-case. Given its therapeutic relevance, approaches to formulate nitazoxanide cocrystals will be of benefit to improve its efficacy, considering that its low solubility impacts its bioavailability. Importantly, nitazoxanide has been proposed as a potential agent for public health control [46].

## 2. Materials and Methods 

### 2.1. Materials

Nitazoxanide was kindly donated by Laboratories Senosiain, S.A de C.V., CDMX, México. Glutaric acid, succinic acid, hydroxypropyl methylcellulose (HPMC, MW: 26 kDa. This product has a methoxyl content of 19–24% and a hydroxypropoxyl content of 7–12%, viscosity 80–120 cP, 2% in H_2_O at 20 °C), methylcellulose (Methocel^®^ 60 HG and Methocel^®^ MC), hydroxypropyl cellulose (HPC 80,000 and HPC 370,000), polyvinylpyrrolidone (Kollidon^®^ 25), polyethylene glycol (Kollisolv^®^) and poly(ethylene glycol)-block-poly(propylene glycol)-block-poly(ethylene glycol) (Kolliphor^®^ P 407) were purchased from Sigma-Aldrich (St. Louis, MO, USA) as reagents for use only in the laboratory. All organic solvents used were purchased from J. T. Baker Chemical Co. (Phillipsburg, NJ, USA). Solvents and chemical compounds were used as received without further purification.

### 2.2. Methods 

#### 2.2.1. Preparation of NTZ Cocrystals 

NTZ-GLU and NTZ-SUC cocrystals of 1:1 and 2:1 NTZ:coformer stoichiometric ratio, respectively, were synthetized by the liquid-assisted grinding (LAG) technique as previously reported [8]. Samples of 1.0 g were ground in a Retsch MM400 mixer (Haan, Germany; 0.5 h at 25 Hz) with acetone. The formation of the cocrystalline solid phases was established by comparison of the powder X-ray diffraction (PXRD) patterns with both experimental and simulated patterns obtained from reported X-ray crystal structures (REFCODES: BREEINM and BRENVIEX).

#### 2.2.2. Solvent-Shift Method

A total of eight candidate polymers were evaluated to test their ability to maintain NTZ solubilized: Methocel^®^ MC, Methocel^®^ 60 HG, HPMC, HPC 80,000, HPC 370,000, Kollisolv^®^, Kolliphor^®^ P 407, and Kollidon^®^ 25 (see Table S1, for further polymer details). The tests were performed by the modified solvent shift method proposed by Childs et al. in 2013 [24], see Figure S1. Aqueous solutions were prepared containing 0.5% *w*/*v* of each pre-dissolved polymer in pH 7.5 phosphate buffer solution (PBS) and also one containing only buffer solution. A volume of 3 mL of each medium was placed in a spectrophotometer quartz cell, followed by a stepwise addition of a total of 13 aliquots of 10 µL each from NTZ stock solution in DMSO (25 mg/mL) at 5 min interval times. The ability of the polymeric compounds to maintain the API dissolved was determined by analysis of the presence or absence of precipitated NTZ in solution, which can be monitored by UV-vis spectrophotometry measured at *λ* = 550 nm. 

#### 2.2.3. Powder Dissolution under Non-Sink Conditions

For the powder dissolution experiments under non-sink conditions, 200 mg of pure NTZ or the equivalent of NTZ cocrystal were added to 10 mL of pH 7.5 buffer dissolution medium. All NTZ samples were passed through a sieve mesh 200.

The dissolution experiments were carried out at 37 ± 0.5 °C with magnetic stirring at 90 rpm (Personal Reaction Station, J-Kem Scientific Inc., St. Louis, MO, USA). Samples of 1 mL were withdrawn and subsequently filtered (Whatman 3), each minute during the first 10 min, then every two minutes until 30 min, and finally at 45, 60, 75 and 90 min. A total of 200 µL samples were then diluted to 5 mL with the corresponding dissolution medium and analyzed by UV-vis spectrophotometry at 435 nm to determine the amount of NTZ dissolved. Dissolution test with solutions containing pre-dissolved HPMC or Methocel^®^ 60 HG polymer (0.5% *w*/*v*) in pH 7.5 PBS were employed and compared with the results obtained from polymer-free solutions (PBS alone). All experiments were carried out in triplicate. After the dissolution experiments, the solid residues were collected and dried at room temperature for analysis by powder X-ray diffraction (PXRD).

#### 2.2.4. Preparation of Powder Formulations with Methocel^®^ 60 HG

Powder formulations with NTZ consisted in physical mixtures of the components listed in Table 1. Formulations at three different concentrations (1.0, 2.5 and 5.0% *w*/*w*) of Methocel^®^ 60 HG were prepared considering 250 mg of nitazoxanide in the powder mix (entries 4 to 12). For comparison, the corresponding control experiments in the absence of Methocel^®^ 60 HG were also obtained (entries 1, 2 and 3). All NTZ samples were passed through a sieve mesh 200.

#### 2.2.5. Powder Dissolution Experiments in the USP 1 (Basket) Apparatus

For the dissolution experiments employing the USP 1 apparatus, the powder formulations shown in Table 1 were added to a volume of 600 mL of pH 7.5 PBS solution, with a rotation speed of 100 rpm at 37 ± 0.5 °C. Samples of 4 mL were taken at 2, 5, 10, 15, 30, 60, 120 and 180 min, and an equal volume of fresh medium was added to maintain the dissolution medium volume constant. The NTZ concentration was quantified by UV-vis spectrophotometry at 435 nm (Figures S2 and S3). Each dissolution profile represents the average of three experiments. After the dissolution experiments, the solid residues were collected and dried at room temperature for analysis by PXRD and by scanning electron microscopy (SEM). Finally, a test comparing a powder formulation with Methocel^®^ 60 HG 5% *w*/*w* containing 500 mg of NTZ as NTZ-SUC cocrystal and a commercially available formulation (500 mg tablet) was performed. The reference tablets of NTZ (Daxon^®^, 500 mg NTZ) were ground in an agate mortar and passed through a sieve mesh 200. All the dissolution profiles are presented as mg of NTZ dissolved vs. time. The mg of NTZ dissolved was calculated as:

$$\mathrm{mg}\;\mathrm{of}\;\mathrm{drug}\;\mathrm{dissolved}=C_{n}\times V_{n}+\sum_{i=1}^{n-1}C_{i}\times V_{s}$$

where *C_n_* = concentration of drug in sample *n*, *V_n_* = volume of dissolution media at the time of taking the sample *n*; *C_i_* = concentration of drug in sample *n* − 1 and *V_s_* = volume of aliquot due to sampling [47].

### 2.3. Characterization Techniques

#### 2.3.1. Powder X-Ray Diffraction Analysis (PXRD)

The solid phases resulting from the NTZ cocrystal synthesis and the samples recovered from the powder dissolution experiments were examined by PXRD analysis using a BRUKER D8-ADVANCE diffractometer (Bruker, Bremen, Germany) equipped with a LynxEye detector (*λ*_Cu*K*α1_ = 1.5406 Å, monochromator: germanium). The equipment was operated at 40 kV and 40 mA, and data were collected at room temperature in the range of 2*θ* = 5–45°. Powder diffraction patterns were compared with the simulated patterns from single crystal X-ray diffraction (SCXRD) data.

#### 2.3.2. Scanning Electron Microscopy (SEM) Analysis

The solid residuals obtained after the dissolution tests under sink conditions were analyzed by SEM. For this purpose, the samples were coated with a gold layer, where after SEM micrographs were captured by a VEGA 3 TESCAN scanning electron microscope (Tescan Orsay Holdings, Kohoutovice, Czech Republic) at 10.0 kV.

### 2.4. Statistical Analysis 

The differences in the dissolution profiles were analyzed by two-way variance analysis (ANOVA) of the area under the curve (AUC), using the Origin Pro 9.0 software package (OriginLab Co., Northampton, MA, USA). Subsequently, a multiple post hoc test (Tukey) was performed with a significance level of 0.05 using the Statgraphics software (Statgraphics Technologies, Inc., The Plains, VA, USA).

## 3. Results

### 3.1. Preparation and Characterization of NTZ Cocrystals 

NTZ was combined with GLU and SUC in 1:1 and 2:1 stoichiometric ratio, respectively, using the liquid-assisted grinding method with acetone as solvent as previously reported [8]. NTZ-SUC and NTZ-GLU cocrystals were prepared in scales of 1.0 g, and in all cases a homogeneous single phase was obtained, for which the experimental PXRD pattern agreed with the pattern simulated from the crystal structure determined by single-crystal X-ray diffraction analysis (see Figure S4). A batch of ten (individually characterized) samples was finally mixed and used for all subsequent experiments.

### 3.2. Polymer Selection by Solvent-Shift Method

**Figure 1:**
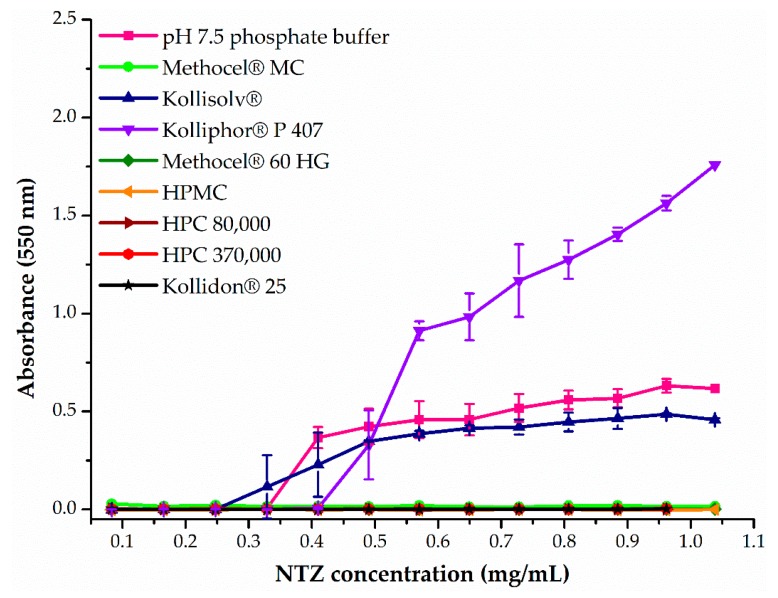
Inhibitory effect of pre-dissolved polymers (0.5% *w*/*v*) on NTZ precipitation in pH 7.5 PBS. (*n* = 3 ± SD).

Figure 1 shows the effect of polymers commonly employed in pharmaceutical drug formulations to inhibiting or delaying precipitation of NTZ. In the absence of polymers, addition of aliquots from NTZ stock solution (25 mg/mL) to 3 mL of a phosphate buffer solution (PBS, pH 7.5) caused precipitation of NTZ at concentrations above 0.30 mg/mL. In the presence of 0.5% *w*/*v* pre-dissolved cellulosic polymers, i.e., Methocel^®^ MC, Methocel^®^ 60 HG, HMPC, HPC 80,000 and HPC 370,000, as well as the polyvinylpyrrolidone Kollidon^®^ 25, precipitation of NTZ was inhibited, reaching concentrations of at least 1.0 mg/mL; meanwhile, polyethylene glycol Kollisolv^®^ and poloxamer Kolliphor^®^ P 407 maintained the drug dissolved only at concentrations below 0.25–0.40 mg/mL. Based on these results, HPMC and Methocel^®^ 60 HG were selected for subsequent powder dissolution experiments of NTZ and the cocrystal phases with GLU and SUC under non-sink conditions.

### 3.3. Powder Dissolution under Non-Sink Conditions 

**Figure 2:**
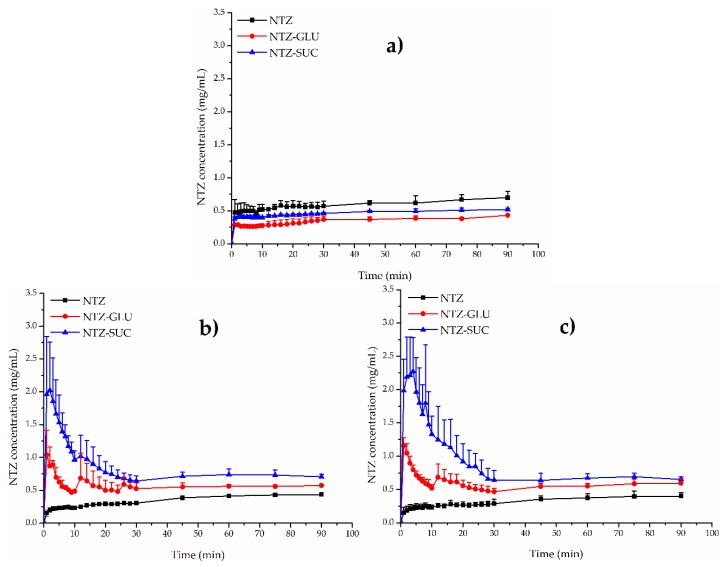
Powder dissolution profiles of NTZ and the cocrystalline phases NTZ-GLU and NTZ-SUC in: (**a**) pH 7.5 PBS; (**b**) PBS with pre-dissolved HMPC (0.5%, *w*/*v*); and (**c**) PBS with pre-dissolved Methocel^®^ 60 HG (0.5%, *w*/*v*) (*n* = 3 ± SD).

**Figure 3:**
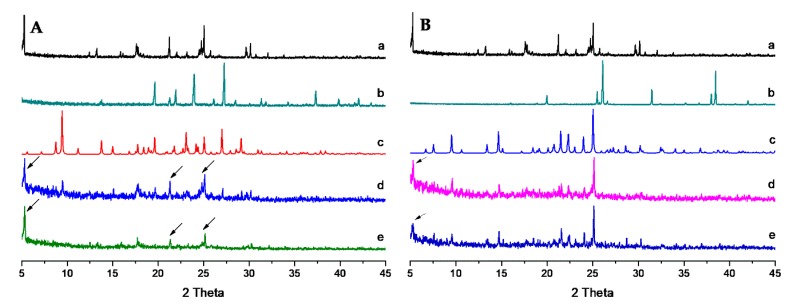
Comparison of powder X-ray diffraction (PXRD) patterns: (**A**) (a) NTZ, (b) GLU, (c) NTZ-GLU simulated from SCXRD data. Solids recovered after powder dissolution tests of NTZ-GLU under non-sink conditions in pH 7.5 phosphate buffer solution after (d) 2 min, and, (e) 5 min. (**B**) (a) NTZ, (b) SUC, (c) NTZ-SUC simulated from SCXRD data. Solids recovered after powder dissolution tests of NTZ-SUC under supersaturated conditions in pH 7.5 phosphate buffer solution after (d) 1 min, and (e) 3 min.

**Figure 4:**
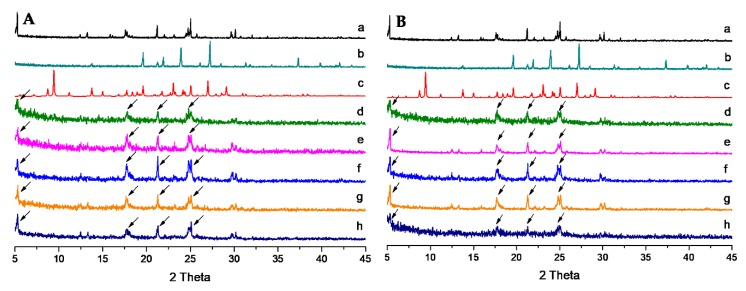
Comparison of PXRD patterns: (**A**) (a) NTZ, (b) GLU, (c) NTZ-GLU simulated from SCXRD data. Solids recovered after powder dissolution tests of NTZ-GLU under non-sink conditions in pH 7.5 phosphate buffer solution with pre-dissolved 0.5% *w*/*v* HPMC after (d) 1 min, (e) 2 min, (f) 3 min, (g) 4 min, (h) 5 min (**B**) (a) NTZ, (b) GLU, (c) NTZ-GLU simulated from SCXRD data. Solids recovered after powder dissolution tests of NTZ-GLU under non-sink conditions in pH 7.5 phosphate buffer solution with pre-dissolved 0.5% *w*/*v* Methocel^®^ 60 HG after (d) 1 min, (e) 2 min, (f) 3 min, (g) 4 min, (h) 5 min.

**Figure 5:**
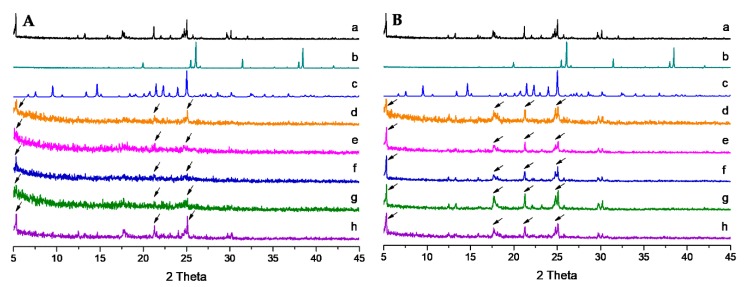
Comparison of PXRD patterns: (**A**) (a) NTZ, (b) SUC, (c) NTZ-SUC simulated from SCXRD data. Solids recovered after powder dissolution tests of NTZ-SUC under non-sink conditions in pH 7.5 phosphate buffer solution with pre-dissolved 0.5% *w*/*v* HPMC after (d) 1 min, (e) 2 min, (f) 3 min, (g) 4 min, (h) 5 min. (**B**) (a) NTZ, (b) SUC, (c) NTZ-SUC simulated from SCXRD data. Solids recovered after powder dissolution tests of NTZ-SUC under non-sink conditions in pH 7.5 phosphate buffer solution with pre-dissolved 0.5% *w*/*v* Methocel^®^ 60 HG after (d) 1 min, (e) 2 min, (f) 3 min, (g) 4 min, (h) 5 min.

Powder dissolution profiles of pure NTZ and the NTZ-GLU/NTZ-SUC cocrystals were measured in pH 7.5 phosphate buffer solution in the absence and presence of pre-dissolved polymer (0.5%, *w*/*v*; 5 mg/mL). The corresponding graphs are shown in Figure 2. In the absence of polymer, there is no statistically significant difference (*p* > 0.05) between the profile of NTZ and those obtained for the cocrystals (Figure 2a), maintaining on average a saturated NTZ concentration of 0.5 mg/mL. On the contrary, in the presence of pre-dissolved HPMC and Methocel^®^ 60 HG, the NTZ dissolution profiles of the pure form and the cocrystals are significantly different (*p* < 0.05) (Figure 2b,c). Thus, starting from the cocrystals, pre-dissolved HPMC and Methocel^®^ 60 HG enable the generation of transient drug concentrations (approximately 2 mg/mL) that are 4 times above NTZ solubility, following a spring–parachute profile as postulated by Guzman et al. [48]. This supersaturation state is sustained for approximately 30 min, for both cocrystals. The decay of the NTZ concentration is attributed to precipitation of pure NTZ, as indicated by PXRD analyses of the solid residues recovered after the dissolution experiments with NTZ-GLU and NTZ-SUC, that showed characteristic diffraction peaks of pure NTZ (Figure 3, Figure 4 and Figure 5). Overall, this analysis shows that indeed NTZ-GLU and NTZ-SUC have superior dissolution properties compared to the parent drug, suggesting that dissolution kinetics of the cocrystalline phases is extremely rapid, supersaturating the solution and precipitating in a short time period (~1–5 min), which is in agreement with our previous studies on the solution-phase stability of NTZ-GLU and NTZ-SUC in PBS pH 7.5 [8]. However, in the presence of pre-dissolved HPMC and Methocel^®^ 60 HG, the precipitation of pure NTZ is delayed by a time interval long enough to envision a potential improvement of the bioavailability (~30 min, see Discussion section).

### 3.4. USP 1 Apparatus Powder Dissolution Experiments of Formulations with Polymer

**Figure 6:**
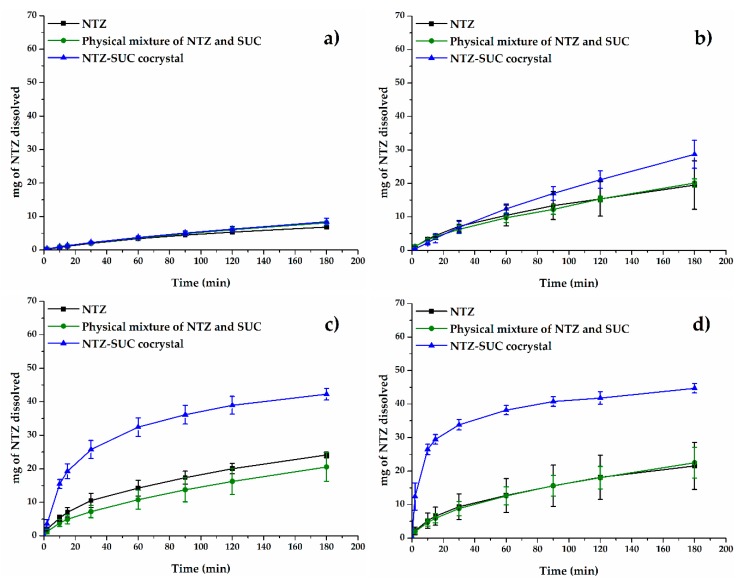
Powder dissolution profiles (in pH 7.5 PBS at 37 ± 0.5 °C) for pure NTZ, the 2:1 stoichiometric physical mixture of NTZ and SUC, and NTZ-SUC (2:1) cocrystal formulated with Methocel^®^ 60 HG (*w*/*w*): (**a**) 0.0%; (**b**) 1.0%; (**c**) 2.5%; (**d**) 5.0% (*n* = 3 ± SD).

The above-described powder dissolution experiments under non-sink conditions showed that the best improvement of the NTZ dissolution profile was achieved with the NTZ-SUC cocrystal, in the presence of Methocel^®^ 60 HG. To evaluate if the polymer included in the solid formulation has an effect on the dissolution kinetics of the cocrystal, four pharmaceutical powder formulations were examined with cocrystal–polymer ratios of 0.0, 1.0, 2.5 and 5.0% (*w*/*w*). The dissolution performance of the formulated solid samples was analyzed by means of powder dissolution tests using the USP 1 (basket) apparatus. A similar strategy was employed for the evaluation of formulated cocrystals of carbamazepine [29,30]. The dissolution profiles in pH 7.5 PBS for NTZ in pure form, a 2:1 stoichiometric physical mixture of NTZ and SUC, and NTZ-SUC (2:1) cocrystals formulated with an increasing amount of Methocel^®^ 60 HG are given in Figure 6.

Figure 6a illustrates the dissolution profiles measured over a time interval of 3 h of the unformulated (0.0% *w*/*w* of Methocel^®^ 60 HG) solid phases of pure NTZ, the physical mixture with SUC and the NTZ-SUC cocrystal. The drug dissolution profiles are similar (*p* > 0.05), achieving approximately 8 mg of dissolved drug (ca. 3% in relation to 250 mg of NTZ dose). In contrast, when the solid phases were formulated with 1.0% Methocel^®^ 60 HG, the drug was dissolved in significantly larger amounts, either starting from the pure form, the physical mixture or the cocrystal (~17–27 mg of drug dissolved after 3 h). Although the graph associated to NTZ-SUC exhibits a larger increase, the statistical difference (*p* > 0.05) between the dissociation rates is not significant due to the relatively large standard deviation (Figure 6b). However, with larger amounts of Methocel^®^ 60 HG polymer (2.5 and 5.0%), the NTZ-SUC cocrystal gave a significantly improved dissolution profile (*p* < 0.05) when compared to analogous formulations of pure NTZ and the physical mixture, achieving after 3 h, amounts of 42 and 45 mg of NTZ dissolved (Figure 6c,d). Furthermore, for the formulation of the cocrystal with 5% Methocel^®^ 60 HG, the amount of NTZ dissolved after 2 min was already approximately 12 mg (Figure 6d), which is significantly larger than the final amount achieved by the corresponding unformulated solid phases (Figure 6a).

**Figure 7:**
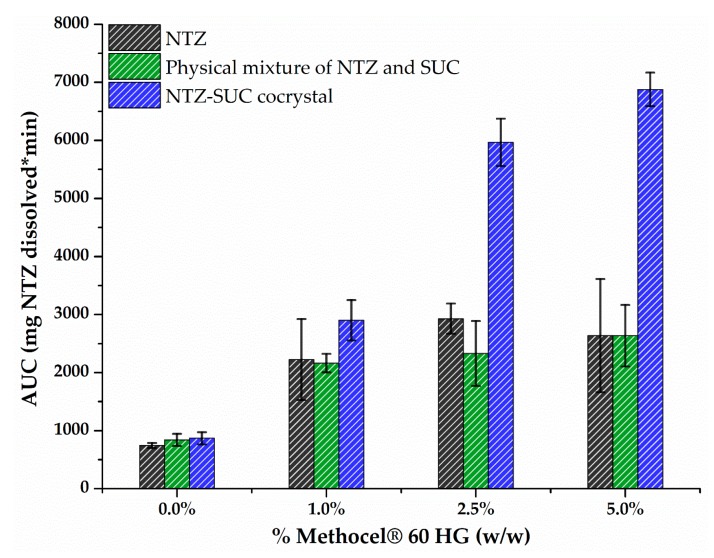
AUC of NTZ extracted from the dissolution profiles in pH 7.5 PBS for the following solid phases, which were formulated with different amounts of Methocel^®^ 60 HG (0.0, 1.0, 2.5 and 5.0%, *w*/*w*): NTZ, 2:1 physical mixture of NTZ and SUC, and NTZ-SUC cocrystal.

The cocrystal solubilization can be quantified by the AUC (area under the curve), thus constituting a parameter indicative of the influence that cocrystals have on the API performance. The AUC is directly and inversely proportional to the dissolution and precipitation rates, respectively [49]. Figure 7 shows that unformulated NTZ-SUC cocrystals and NTZ-SUC formulated with 1.0% *w*/*w* Methocel^®^ 60 HG do not lead to improved dissolution profiles when compared to NTZ and the physical mixture of NTZ and SUC (*p* > 0.05). However, for the formulations with 2.5 and 5.0% (*w*/*w*) of polymer, the AUC for the NTZ-SUC cocrystal increased by factors of 8.0 and 9.2, respectively, in comparison to the unformulated NTZ. Meanwhile, for NTZ and the physical mixture with SUC the AUC are practically constant in the polymer formulations examined herein.

**Figure 8:**
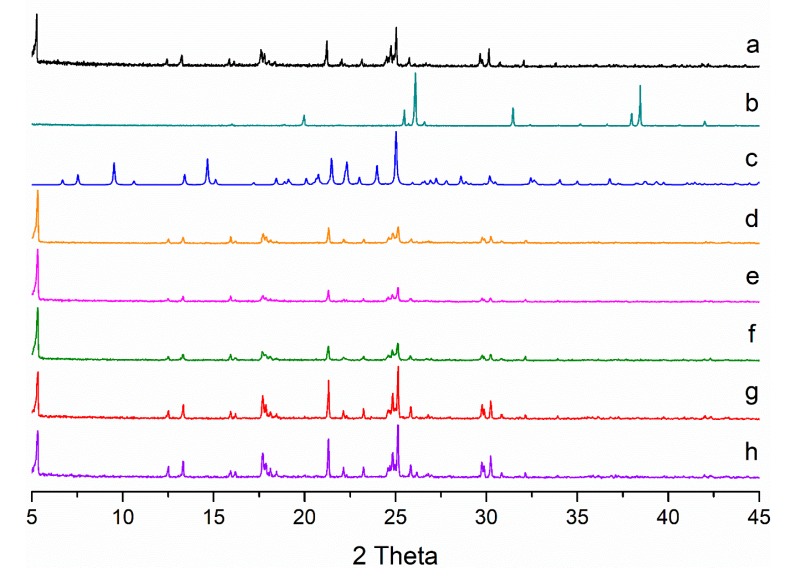
PXRD patterns of (a) NTZ, (b) SUC, (c) NTZ-SUC cocrystal (simulated from SCXRD data) and solid residues after dissolution tests in USP 1 apparatus: (d) NTZ-SUC formulated with 1.0% *w*/*w* Methocel^®^ 60 HG, (e) NTZ-SUC formulated with 2.5% *w*/*w* Methocel^®^ 60 HG, (f) NTZ-SUC formulated with 5.0% *w*/*w* Methocel^®^ 60 HG, (g) NTZ formulated with 5.0% *w*/*w* Methocel^®^ 60 HG, and (h) physical mixture of NTZ and SUC formulated with 5.0% *w*/*w* Methocel^®^ 60 HG.

Selected samples of the solid residues recovered after the dissolution tests were analyzed by PXRD and SEM analysis. The PXRD data showed that the NTZ-SUC cocrystal is transformed into its parent drug in all of the formulations tested (Figure 8), and it was also observed in the dissolution experiments under non-sink conditions. Peaks for SUC are not observed, indicating that the coformer was completely dissolved, which is in agreement with its good solubility in water (71 mg/mL at 25 °C).

**Figure 9:**
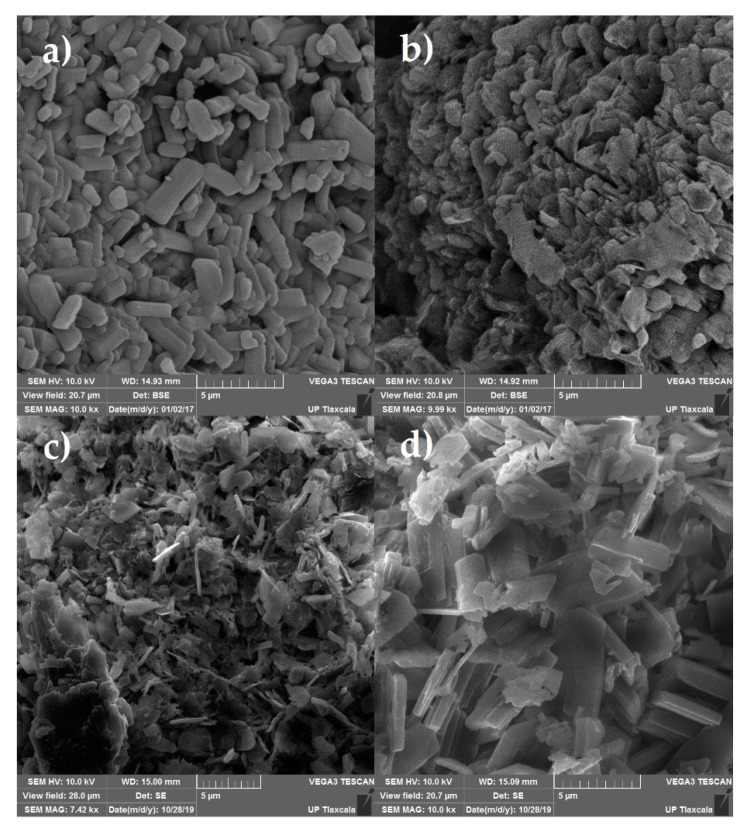
SEM images of (**a**) NTZ-SUC cocrystals before the powder dissolution test, and the solid residues recovered after the dissolution experiments in the USP 1 apparatus: NTZ-SUC cocrystal formulated with (**b**) 1.0%, (**c**) 2.5%, and (**d**) 5% of Methocel^®^ 60 HG.

Figure 9 presents SEM images of the cocrystal phase NTZ-SUC before and after the dissolution experiments in the presence of different amounts of Methocel^®^ 60 HG. Freshly prepared NTZ-SUC cocrystals exhibit a characteristic and homogeneous cylindrical morphology with a small particle size (Figure 9a) in comparison with pure NTZ and the physical mixture with SUC (Figure S5a,b). The SEM images of the solid residues recovered after the dissolution tests of NTZ and the physical mixture formulated with Methocel^®^ 60 HG at 1.0 and 5.0% (*w*/*w*) show slight changes of the morphology in the presence of polymer (Figure S5c–f). A more pronounced effect occurs for the NTZ-SUC cocrystals with the morphology of the samples changing significantly with increasing amount of polymer (Figure 9b–d).

**Figure 10:**
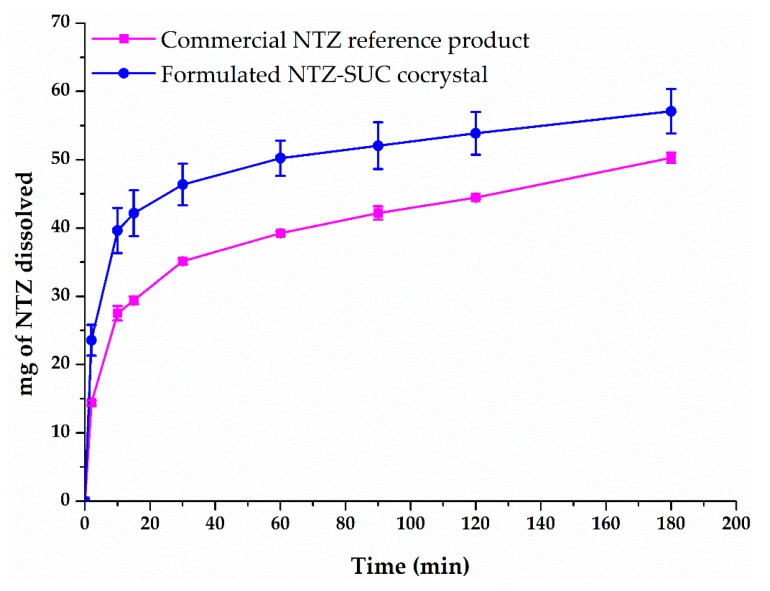
Powder dissolution profiles of a commercially available formulated NTZ medicine and the formulated powder of NTZ-SUC cocrystals with Methocel^®^ 60 HG (5% *w*/*w*) in pH 7.5 PBS at 37 ± 0.5 °C (*n* = 3 ± SD).

Because of the promising results, the performance of the powder formulation of NTZ-SUC cocrystal with Methocel^®^ 60 HG (5% *w*/*w*) was compared with a commercially available formulation of NTZ. Since our studies were based on a pharmaceutical powder, the commercially available reference tablets of NTZ (Daxon^®^, 500 mg NTZ) were ground in an agate mortar and sifted through a sieve mesh 200. The results of the powder dissolution experiments using the USP type 1 apparatus (following the protocol detailed in Section 2.2.5) are shown in Figure 10. Comparison of the dissolution graphs shows a statistically significant improvement of the dissolution properties for the formulated NTZ-SUC cocrystal in comparison with the commercial NTZ medicine (*p* < 0.05). From the polymer–cocrystal formulation with Methocel^®^ 60 HG (5% *w*/*w*), 57 mg of the drug were dissolved in the bulk solution after 3 h with an amount dissolved of 9046 ± 533 mg NTZ*min (AUC_0–3 h_), while the commercial product achieved 50 mg in the same time period, with an amount of 7287 ± 77 mg NTZ*min dissolved.

## 4. Discussion

In a recent review Zaworotko and coworkers enlisted a total of eight commercially available pharmaceutical cocrystals [50], showing that cocrystalline solid forms of current APIs are adequate for the fabrication of medicines for various types of therapies. At the same time, in the last few years the number of publications and patents related to pharmaceutical cocrystals has been increasing [50,51]. Nevertheless, there are still several challenges for the development of cocrystals in pharmaceutical solid dosage forms, particularly in controlling the behavior of cocrystal dissolution, drug supersaturation and precipitation kinetics, which can be achieved by selecting suitable excipients that will delay nucleation and/or crystallization, and maintain adequate drug supersaturation levels [13,51].

In the research project presented herein, two cellulosic polymers were chosen to evaluate their effect on maintaining the supersaturated state achieved during the dissolution of cocrystalline solid phases of nitazoxanide, a poorly soluble drug. Subsequently, polymer–cocrystal powder formulations were developed. To select adequate candidates from the series of widely used polymers in the pharmaceutical industry that delay the nucleation and/or crystallization of NTZ in supersaturated conditions, the solvent-shift approach was used [24], see Figure S1. Figure 1 demonstrates the results with the polymers tested for NTZ in pH 7.5 PBS at room temperature, finding that non-cellulosic polymers (Kollisolv^®^ and Kolliphor^®^ P407) do not significantly help in delaying NTZ precipitation, while polyvinylpyrrolidone (PVP-Kollidon^®^ 25) and cellulosic polymers (HPC 80,000, HPC 370,000, HPMC, Methocel^®^ MC and Methocel^®^ 60 HG) enable the increase of the concentration of NTZ in aqueous solution up to at least 1.0 mg/mL. Considering the hydrogen bonding capacity, NTZ has one H-bond donor group (NH) and four H-bond acceptor sites [8]. As can be observed in Scheme 1, the cellulosic polymers HPMC and Methocel^®^ 60 HG contain a large number of O–H donor groups, which are complementary for the formation of hydrogen bonds with hydrogen bond acceptors, explaining the good precipitation inhibitor properties for NTZ. The remaining polymers tested herein lack strong H-bond donor functions that could interact through hydrogen bonds with NTZ. These findings are supported by previous reports in the literature, e.g., in 2010, Warren et al. described the effectiveness of cellulosic polymers as crystallization inhibitors for danazol [52]. Other reports showed that HPMC reduces efficiently the nucleation and crystal growth of felodipine [53] and celecoxib [54] under non-sink conditions. Moreover, it is reasonable to assume that cellulose derivatives do inhibit, not only nucleation but also crystal growth and agglomeration into larger sized particles (see SEM results) that otherwise may form from during NTZ precipitation. In other words, the precipitation delay of NTZ can also be attributed to surface interactions between the NTZ particles and the polymer, forming drug-polymer complexes, which is derived in the modification of the NTZ solubilization properties.

### 4.1. Performance of NTZ Cocrystals under Non-Sink Conditions

Based on the large solubility difference between NTZ and aliphatic dicarboxylic acids as coformers [8], we expected that cocrystal forms of NTZ with GLU and SUC increase the aqueous solubility of NTZ. Figure 2a shows that NTZ-GLU and NTZ-SUC cocrystals have dissolution profiles similar to NTZ in pH 7.5 phosphate buffer solution, and a higher solubilization of NTZ is not observed under these conditions. Indeed, rapid conversion of the cocrystals into pure drug was demonstrated by PXRD analysis of the solids recovered after the dissolution tests in short time periods (~1–5 min, Figure 3). A similar behavior has been documented for other cocrystal phases in the literature [24,25,30,36,37,55,56,57]. For example, exemestane–maleic-acid cocrystals did not increase solubilization of the drug due to rapid phase transformation [55]. In a recent publication this cocrystal was reinvestigated observing that transformation into the parent drug occurred in less than 1 min in fasted state simulated intestinal fluid (FaSSIF) [37]. The critical role of the coformer physicochemical properties on the solubility and dissolution advantages of cocrystals it is now accepted, even though the exact mechanism by which this enhancement occurs is not fully understood. In NTZ cocrystals studied here, the coformers GLU and SUC are ionizable compounds (p*K*_a_ values of 4.3 for GLU and 4.2 for SUC) and exhibit favorable water solubilities of 540 mg/mL and 71 mg/mL at 25 °C, respectively [12,58]. The fast dissolution–supersaturation phenomenon induced for the NTZ cocrystals under the experimental conditions used herein (pH 7.5 PBS), is attributed to the favorable solubilization of these coformers. This rapid cocrystal dissolution produces drug supersaturation levels that are difficult to control in absence of suitable excipients at adequate concentrations, and therefore NTZ solubilization advantage was not observed.

The use of excipients such as polymers and surfactants constitutes an efficient strategy to prolong drug supersaturation achieved from pharmaceutical cocrystals [23,24,25,36,57]. An addition of a cellulosic polymer into the dissolution medium for powder dissolution studies under non-sink conditions revealed that these can induce a supersaturation effect starting from NTZ-SUC and NTZ-GLU cocrystals. Both Methocel^®^ 60 HG and HPMC were able to maintain drug supersaturation (up to 4 times higher concentrations of NTZ in solution over solubility value) for a period long enough to display a superior dissolution profile (Figure 2b,c). The phenomenon was upheld for up to 30 min, emulating the spring–parachute effect [48]. There are some studies demonstrating an in vitro/in vivo correlation of formulated cocrystals. In these cases, the in vitro dissolution test showed that the high drug concentration achieved by cocrystalline phases is maintained by 30–40 min and the bioavailability of the corresponding poorly soluble drugs was enhanced [24,33]. Considering these experimental data, it is considered that the increase of NTZ dissolved from NTZ cocrystals formulated with polymers may promote NTZ absorption.

The physical stability of the NTZ cocrystals used in the powder dissolution experiments was monitored by PXRD analysis, revealing that rapid cocrystal dissolution followed by drug precipitation still occurred in the presence of the polymer in solution (Figure 4 and Figure 5). After 1 min in contact with pH 7.5 PBS, the powders analyzed by PXRD analysis indicated complete transformation to pure NTZ, and the patterns did not exhibit peaks indicating the presence of coformer in the solid.

Cellulosic polymers are the most common hydrophilic polymers used for the development of formulations with cocrystals [24,26,28,29,33,36,37]. They can inhibit or at least delay phase transformations of cocrystals [29,30], by establishing drug-polymer interactions [37] and maintaining the solubilization state of APIs. Therefore, the improvement of the NTZ dissolution profiles in the presence of pre-dissolved polymer could be explained by the following mechanism: after the rapid NTZ release into solution starting from the cocrystal, the cellulosic polymer interacts with NTZ molecules delaying its nucleation and eventually NTZ crystallized also interact with polymer interfering the crystal growth and agglomeration process.

### 4.2. NTZ-SUC Cocrystal Formulation vs. Formulation with Pure NTZ

Previous formulations with cocrystals were based on polymer matrix tablets [29,30] and pre-dissolved polymers [24,36]. However, there are few studies which have tested the effect of a polymer incorporated in the powder solid-state form on the dissolution profiles of cocrystals [23]. In order to evaluate the effect of Methocel^®^ 60 HG on the dissolution process of NTZ-SUC cocrystals starting from a solid polymer–cocrystal formulation, we examined a series of pharmaceutical powder-based formulations containing different amounts of Methocel^®^ 60 HG (0.0, 1.0, 2.5 and 5.0% *w*/*w*) in comparison to analogous forms with pure NTZ and the physical mixture of NTZ and SUC. In the absence of polymer, all solid phases released similar amounts of NTZ into the solution during the powder dissolution studies using the USP 1 apparatus (Figure 6a) and the NTZ-SUC cocrystal did not show a solubility advantage in comparison with the pure drug and the physical mixture. This observation can be attributed to immediate conversion of the cocrystal phase into NTZ and release of SUC into the solution, in accordance with the dissolution experiments under non-sink conditions. Formulations of pure NTZ, the physical mixture with SUC, and NTZ-SUC cocrystals with 1% (*w*/*w*) of Methocel^®^ 60 HG showed dissolution profiles being similar among each other, but at higher concentrations, i.e., 2.5 and 5.0% (*w*/*w*), a significant increase of the dissolution rate was observed (Figure 6c,d and Figure 7).

Despite the inclusion of polymer, the NTZ-SUC cocrystals were transformed fast into pure NTZ, as revealed by PXRD patterns of solid residues recovered after the dissolution tests (Figure 8). However, the SEM analysis reveals that methylcellulose affects significantly the particle size and morphology of NTZ (Figure 9). The polymer–NTZ interactions probably influence the nucleation process and subsequent crystal growth/agglomeration, thus giving high drug concentration in the dissolution medium over a prolonged time period. Although NTZ-polymer interactions are expected to operate also for the formulations with pure NTZ and the physical mixture, these formulations lack the advantageous initial and immediate dissolution increase of the cocrystal components driven by favorable coformer solubilization.

Finally, comparison of the dissolution profiles of NTZ-SUC cocrystals formulated with methylcellulose at 5.0% *w*/*w* with a commercially available NTZ medicine showed that pharmaceutical products containing co-crystallized APIs are promissory. In such a way, reduction of the NTZ dose might become feasible, which would be a large advantage for the administration of this antiparasitic drug, which requires large doses. However, we are aware that to draw conclusions about this possibility and to assure therapeutic efficacy with reduced adverse effects, in vivo studies are required.

## 5. Conclusions

The results presented herein have shown that NTZ cocrystals can induce a superior dissolution behavior in the presence of hydroxypropyl methylcellulose (HMPC) and methylcellulose (Methocel^®^ 60 HG) in comparison to pure NTZ. Although rapid transformation to the less soluble drug form is observed, the inclusion of polymer in the formulations with NTZ-SUC cocrystals in amounts of 2.5 and 5.0% (*w*/*w*) enabled significantly increasing the dissolution rate of NTZ when compared to pure NTZ and the commercially available drug formulation. The comparative analysis of different powder formulations showed that the amount of polymer in the formulation is an important parameter to consider during cocrystal formulation development for accessing a good solubilization potential for poorly soluble APIs in aqueous solutions.

In conclusion, the use of cocrystals combined with an appropriate selection of pharmaceutical excipients as drug precipitation inhibitors or retardants for crystal growth, such as cellulosic polymers, is a feasible approach to modify the dissolution environment and conditions starting from cocrystals. As a result, the development of a polymer–cocrystal powder formulation is a true alternative to enhance the solubilization of poorly water soluble NTZ and to generate solid dosage forms with better bioavailability and efficacy in the treatment of parasitic and viral diseases, among others.

## Figures and Tables

**Table 1:** Composition (mg) of powder formulations with and without Methocel^®^ 60 HG for NTZ, the physical mixture of NTZ and succinic acid (SUC) (2:1), and the NTZ-SUC cocrystal (2:1) ^a^.

Formulation	1	2	3	4	5	6	7	8	9	10	11	12
NTZ	250			250			250			250		
Phys. Mix. NTZ/SUC		298			298			298			298	
NTZ-SUC cocrystal			298			298			298			298
Methocel^®^ 60 HG ^b^	0	0	0	3.8	3.8	3.8	9.5	9.5	9.5	19	19	19

^a^ 298 mg of NTZ-SUC (2:1) is the molar equivalent of 250 mg of NTZ. ^b^ Percentage (*w*/*w*) of the polymer in the formulation was calculated by taking as reference the average weight of the commercial tablet (ca. 758 mg) containing 500 mg of NTZ. The equivalent weight of the tablet for 250 mg of NTZ corresponds to 379 mg.

## Supplementary Materials

The following data are available online at https://www.mdpi.com/1999-4923/12/1/23/s1: Table S1. General characteristics of polymers used. Figure S1. Scheme of the solvent-shift methodology employed to select polymers that delay nucleation and/or API crystallization. Figure S2. The UV-vis quantification method was specific for NTZ at 435 nm. At 2.97 µg/mL of pure NTZ and both cocrystals, there is no interference by coformers in the quantification method used herein. At other concentration levels of NTZ and coformers, the observation was the same, there was no interference in NTZ quantitation by the coformers presence. Figure S3. UV-vis spectra of samples from powders dissolution of NTZ (left) and NTZ-SUC cocrystal (right) formulated at different concentrations of Methocel^®^ 60 HG. NTZ spectrum did not change in presence of polymer. Figure S4. Comparison of PXRD patterns: A. (a) Nitazoxanide, (b) glutaric acid, (c) NTZ-GLU simulated from SCXRD data, (d–m) Batches No. 1–10 of NTZ-GLU cocrystals prepared in l gram scale by SDG with acetone as solvent. B. (a) Nitazoxanide, (b) succinic acid, (c) NTZ-SUC simulated from SCXRD data, (d–m) Batches No. 1–10 of NTZ-SUC cocrystals prepared in l gram scale by SDG with acetone as solvent. Figure S5. SEM images of the solid starting materials used for the powder dissolution tests: (a) NTZ, (b) physical mixture of NTZ and SUC; and solid residues recovered after the dissolution tests of the solids formulated with Methocel^®^ 60 HG: (c) NTZ with 1.0% *w*/*w*, (d) physical mixture of NTZ and SUC with 1.0% *w*/*w*, (e) NTZ with 5.0% *w*/*w*, (f) physical mixture of NTZ and SUC with 5.0% *w*/*w*. Note: The SEM images of the starting materials are presented at higher resolution than the remaining images (see scale in each image).
