# Supplementary material for: Dissolution Advantage of Nitazoxanide Cocrystals in the Presence of Cellulosic Polymers
Source: Pharmaceutics. 2019 Dec 25;12(1):23. doi: 10.3390/pharmaceutics12010023 (PMC7022799; doi:10.3390/pharmaceutics12010023)
Supplement: Supplementary file 1 [file pharmaceutics-12-00023-s001.pdf]

pharmaceutics

Supplementary Materials: Dissolution Advantage of Nitazoxanide Cocrystals in the Presence of Cellulosic Polymers

Table S1. General characteristics of polymers used.

Polymer	Chemical Structure	Molecular Weight	Viscosity
Hydroxypropyl methylcellulose (HPMC)	Methoxyl content: 19–24% Hydroxypropoxyl content: 7–12%	Approximately 26 kDa	80-120 cP, 2% in water (20 °C) (lit.)
Methylcellulose (Methocel® 60 HG)	28–30% methoxyl basis	Not available	45.4 mPa.s, 2% in water (20 °C)
Methylcellulose (Methocel® MC)	27.5–32% methoxyl basis	Not available	3727 mPa.s, 2% in water
Hydroxypropyl cellulose (HPC 80,000)		Average Mw ~80,000, average Mn ~10,000	250.0–800.0 cP, 10% in water (25 °C)
Hydroxypropyl cellulose (HPC 370,000)		Mw ~370,000	0.5 g/mL at 25 °C (lit.)
Polyvinylpyrrolidone (Kollidon® 25)		Not available	Not available
Polyethylene glycol (Kollisolv®)		Not available	92 mPa.s (20 °C)
Poly(ethylene glycol)-block-poly(propylene glycol)-block-poly(ethylene glycol) (Kolliphor® P 407)	Oxyethylene 71.5–74.9%	Not available	Not available

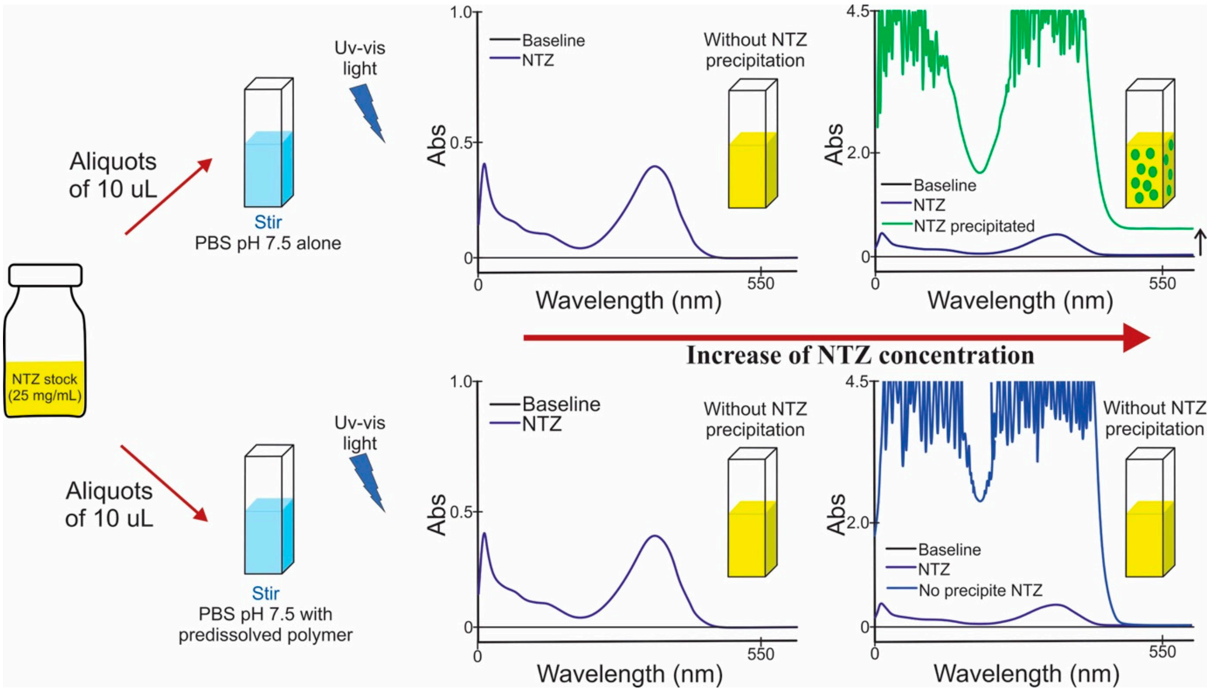

Figure S1. Scheme of the solvent-shift methodology employed to select polymers that delay nucleation and/ or API crystallization.

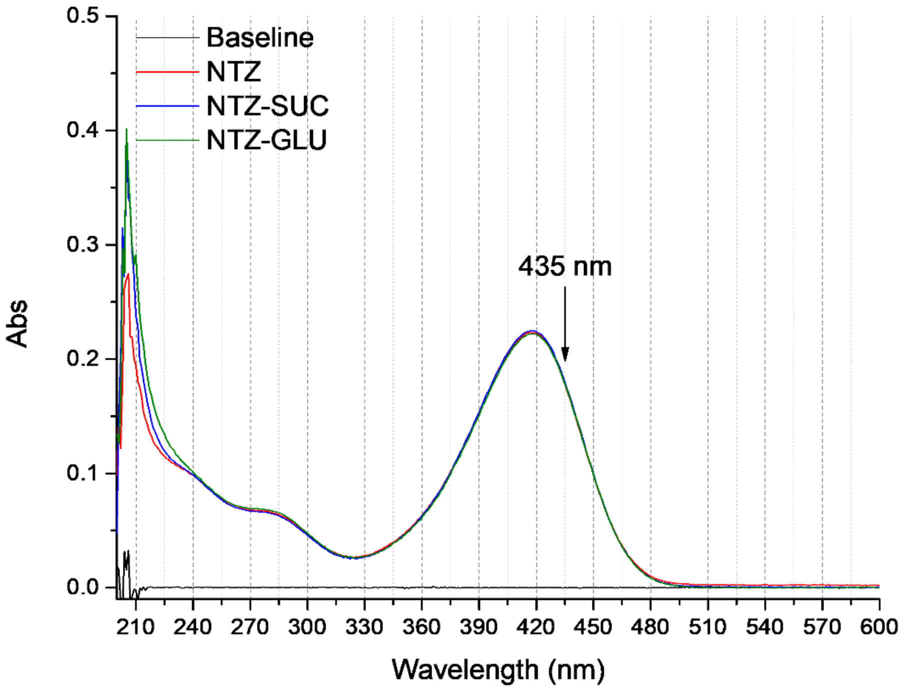

Figure S2. The UV-vis quantification method was specific for NTZ at 435 nm. At 2.97 µg/mL of pure NTZ and both cocrystals, there is no interference by coformers in the quantification method used herein. At other concentration levels of NTZ and coformers, the observation was the same, there was no interference in NTZ quantitation by the coformers presence.

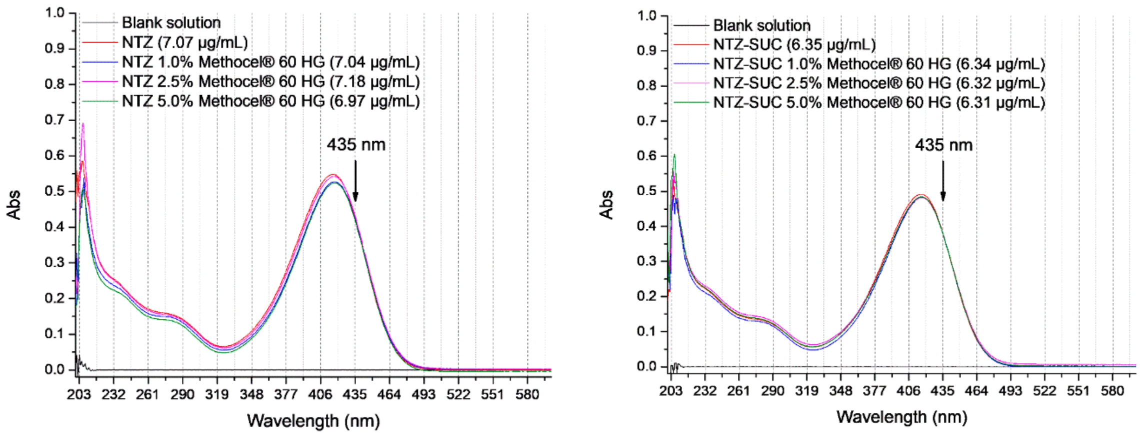

Figure S3. UV-vis spectra of samples from powders dissolution of NTZ (left) and NTZ-SUC cocrystal (right) formulated at different concentrations of Methocel® 60 HG. NTZ spectrum did not change in presence of polymer.

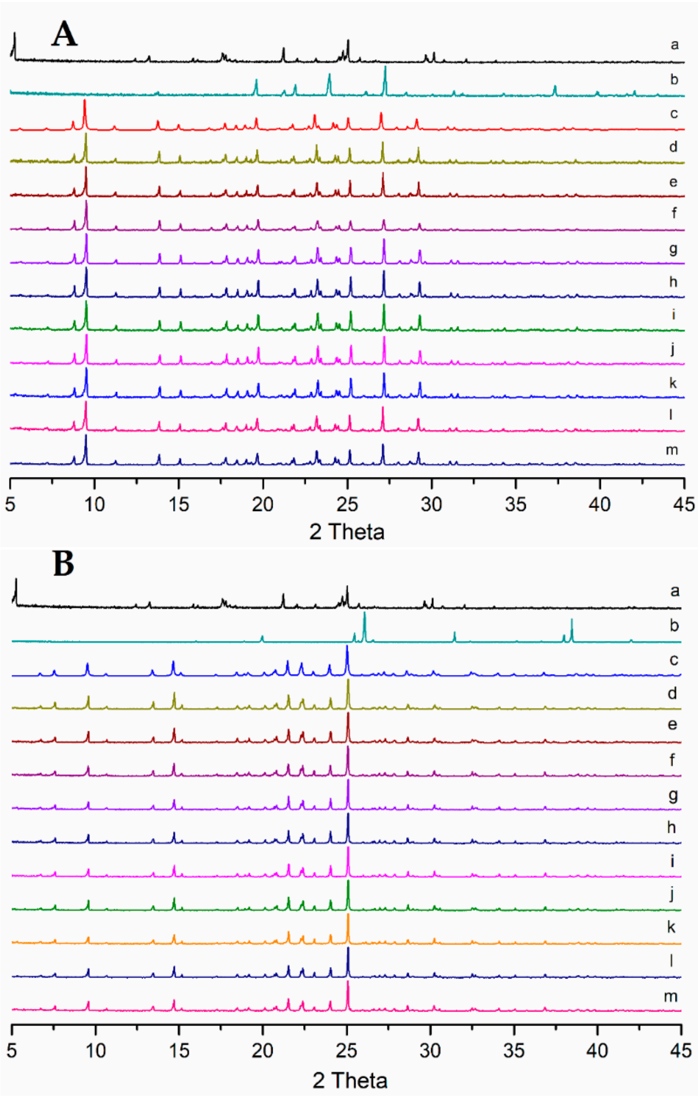

Figure S4. Comparison of PXRD patterns: **A**. a) Nitazoxanide, b) glutaric acid, c) NTZ-GLU simulated from SCXRD data, d)–m) Batches No. 1–10 of NTZ-GLU cocrystals prepared in 1 gram scale by SDG with acetone as solvent. **B**. a) Nitazoxanide, b) succinic acid, c) NTZ-SUC simulated from SCXRD data, d)–m) Batches No. 1–10 of NTZ-SUC cocrystals prepared in 1 gram scale by SDG with acetone as solvent.

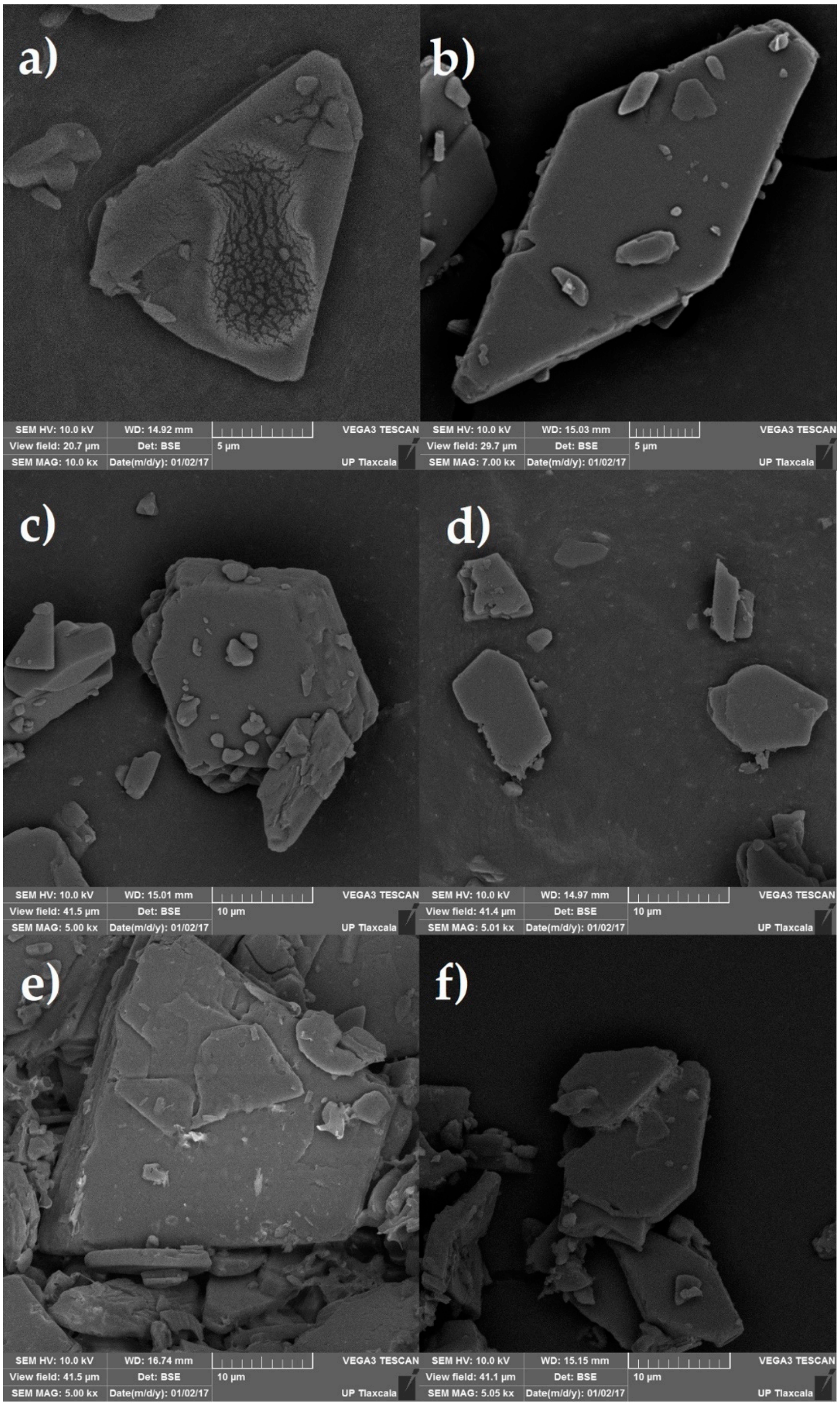

Figure S5. SEM images of the solid starting materials used for the powder dissolution tests: **a**) NTZ, **b**) physical mixture of NTZ and SUC; and solid residues recovered after the dissolution tests of the solids formulated with Methocel® 60 HG: **c**) NTZ with 1.0% *w*/*w*, **d**) physical mixture of NTZ and SUC with 1.0% *w*/*w*, **e**) NTZ with 5.0% *w*/*w*, **f**) physical mixture of NTZ and SUC with 5.0% *w*/*w*. Note: The SEM images of the starting materials are presented at higher resolution than the remaining images (see scale in each image).

© 2019 by the authors. Licensee MDPI, Basel, Switzerland. This article is an open access article distributed under the terms and conditions of the Creative Commons Attribution (CC BY) license (http://creativecommons.org/licenses/by/4.0/).
